# P2P Watch: Personal Health Information Detection in Peer-to-Peer File-Sharing Networks

**DOI:** 10.2196/jmir.1898

**Published:** 2012-07-09

**Authors:** Marina Sokolova, Khaled El Emam, Luk Arbuckle, Emilio Neri, Sean Rose, Elizabeth Jonker

**Affiliations:** ^1^Electronic Health Information LaboratoryCHEO Research InstituteOttawa, ONCanada; ^2^Department of PediatricsFaculty of MedicineUniversity of OttawaOttawa, ONCanada; ^3^Epidemiology and Community MedicineFaculty of MedicineUniversity of OttawaOttawa, ONCanada; ^4^Privacy AnalyticsOttawa, ONCanada

**Keywords:** Privacy, personal health information, natural language processing, text data mining

## Abstract

**Background:**

Users of peer-to-peer (P2P) file-sharing networks risk the inadvertent disclosure of personal health information (PHI). In addition to potentially causing harm to the affected individuals, this can heighten the risk of data breaches for health information custodians. Automated PHI detection tools that crawl the P2P networks can identify PHI and alert custodians. While there has been previous work on the detection of personal information in electronic health records, there has been a dearth of research on the automated detection of PHI in heterogeneous user files.

**Objective:**

To build a system that accurately detects PHI in files sent through P2P file-sharing networks. The system, which we call P2P Watch, uses a pipeline of text processing techniques to automatically detect PHI in files exchanged through P2P networks. P2P Watch processes unstructured texts regardless of the file format, document type, and content.

**Methods:**

We developed P2P Watch to extract and analyze PHI in text files exchanged on P2P networks. We labeled texts as PHI if they contained identifiable information about a person (eg, name and date of birth) and specifics of the person’s health (eg, diagnosis, prescriptions, and medical procedures). We evaluated the system’s performance through its efficiency and effectiveness on 3924 files gathered from three P2P networks.

**Results:**

P2P Watch successfully processed 3924 P2P files of unknown content. A manual examination of 1578 randomly selected files marked by the system as non-PHI confirmed that these files indeed did not contain PHI, making the false-negative detection rate equal to zero. Of 57 files marked by the system as PHI, all contained both personally identifiable information and health information: 11 files were PHI disclosures, and 46 files contained organizational materials such as unfilled insurance forms, job applications by medical professionals, and essays.

**Conclusions:**

PHI can be successfully detected in free-form textual files exchanged through P2P networks. Once the files with PHI are detected, affected individuals or data custodians can be alerted to take remedial action.

## Introduction

Evidence shows that files sent through peer-to-peer (P2P) file-sharing networks can disclose an individual’s personal health information (PHI) to millions of network users. PHI refers to information about one’s health that can be discussed in a clinical setting [1]. For example, in more than 3000 files exchanged on P2P networks, 5% contained either sensitive or sufficient information to commit medical identity theft, sometimes for thousands of individuals [2]. In another study, the authors semimanually examined 859 files gathered from two P2P networks and found that 8 (1%) files contained PHI [3]. Although the disclosure numbers look comparatively small, files on P2P networks are accessible to millions of network users. The same study also showed that the P2P network users may not even be aware that the files can be read by all the peers.

P2P files may be in various media (eg, visual, audio, and text), may address various topics (eg, fashion, tax report, and family life), and may be written in any language (eg, Spanish and French). To effectively deal with such challenges, automated PHI detection must perform well on multiple tasks, such as language identification, filtering out of damaged and virus files, text extraction, and hierarchical multiclass classification of documents. At the same time, the volumes of files exchanged through P2P networks and expectations of privacy of personal communications make manual PHI detection impossible. While there are several traditional PHI detection tools, they are not suitable for the large-volume analysis of heterogeneous documents. For example, some of these tools are designed to work with semistructured electronic health records and to find personal identifiers, such as patients’ and doctors’ names, insurance parameters, and hospital and clinic names [4-10].

In this paper, we describe and evaluate a new system—P2P Watch—that has been constructed specifically to crawl through P2P networks and automatically detect whether a retrieved file contains PHI. To be defined as PHI, a P2P file must contain information that would provide someone with the ability to identify a unique individual as well as health information on that individual, such as procedures or drugs. For example, generic statements such as “John Smith caught a cold” would be rejected as PHI according to our definition, unless they are reinforced by Smith’s residential or work address and the prescription drugs he is taking.

We empirically evaluated our system on three networks: FastTrack, Gnutella, and eD2K. These were chosen due to their global popularity and high share of users. We harvested 3924 files and applied P2P Watch on the file contents. No author metadata was used in the file analysis.

We concentrate on PHI for Canadians. For example, our syntactic patterns that detect provincial health care numbers and the organization types are adjusted for Canada. Although P2P Watch focuses on Canadian PHI, at the same time, it recognizes geographic locations and zip codes in the United States because Canadians may have been born elsewhere or the PHI may concern a trip.

P2P Watch provides a mechanism for data custodians and individuals to determine whether information about their patients, employees, or themselves is being exposed. To minimize potential organizational and individual harm from such inappropriate disclosures, automated PHI detection tools can crawl through P2P networks looking for PHI. Once PHI is detected, affected individuals and data custodians can be alerted to take remedial action.

PHI disclosure on P2P networks is part of a wider trend of PHI presence on the Web [11-14]. PHI appears in electronic news, blogs by health care professionals and military personnel, Web-posted user messages, medical student papers, and personal letters [15-23]. For example, Doing-Harris and Zeng-Treitler [15] extracted health-related terms from messages posted on PatientsLikeMe.com. They manually evaluated the used vocabulary and found 651 health terms that were not yet included in a medical thesaurus. Another study analyzed user requests posted on an involuntary childlessness message board [16]. Blogs written by military servicemen were examined to find descriptions of clinically relevant combat exposure [17]. Lampos and Christianini [18] used Wikipedia’s page on influenza and the UK’s National Health Service website for automated extraction of influenza-like illness markers. They subsequently used the markers to find H1N1-related tweets but did not extract personally identifiable information (PII) about the users. Sokolova et al [19] presented a method of patient-based health information extraction from P2P files. They manually evaluated extraction accuracy on 2000 P2P files.

Interest in PHI published on the Web is ongoing [20]. Although openness and information sharing are beneficial to the population at large, users may not be aware of the secondary use of their information and consequent privacy issues [21]. A qualitative study of 123 user comments on the online community PatientsLikeMe was dedicated to analysis of sharing PHI among people with similar ailments [22]. Chou et al [23] identified younger users, those with poorer subjective health, and those with a personal cancer experience as more likely participants in online support groups and more willing to share their PHI.

So far, PHI detection tools have been developed and deployed by health care organizations in the context of de-identifying the organization’s records, such as clinical discharge summaries, nurses’ notes, and pathology reports. The main de-identification approach was to classify individual words as presenting personally identifiable information (PII) or not [4-10]. Such approaches require a substantial amount of labeled training data (eg, 1000 documents [7]) and consume considerable processing time.

In electronic health records, PHI detection can be boosted by the use of the personal information found in the structured part of the document or by pulling in structured information from the medical record database. Customized dictionaries present another source of accuracy in detecting PHI—these include local geographic names, health care organizations, and patient names [24]. These tools also can determine with certainty that there is health information in the documents they analyze and therefore focus only on the detection of PII. We provide evidence that such tools can fail to identify PHI in free-form textual files.

## Methods

We designed and implemented P2P Watch, which automatically detects P2P network files that contain PHI.

### System Architecture

The system was designed as a pipeline of seven components: (1) duplicate file removal, (2) media content removal, (3) text extractor, (4) language identifier, (5) publishable content identifier, (6) PII detector, and (7) patient-oriented health information detector. Components 1–4 identify and filter out irrelevant files through a shallow analysis, component 5 finds irrelevant files by applying partial content analysis, and components 6 and 7 identify relevant files within the remaining set. At each stage files may be discarded, resulting in fewer and fewer files making it through the pipeline.

**Figure 1:**
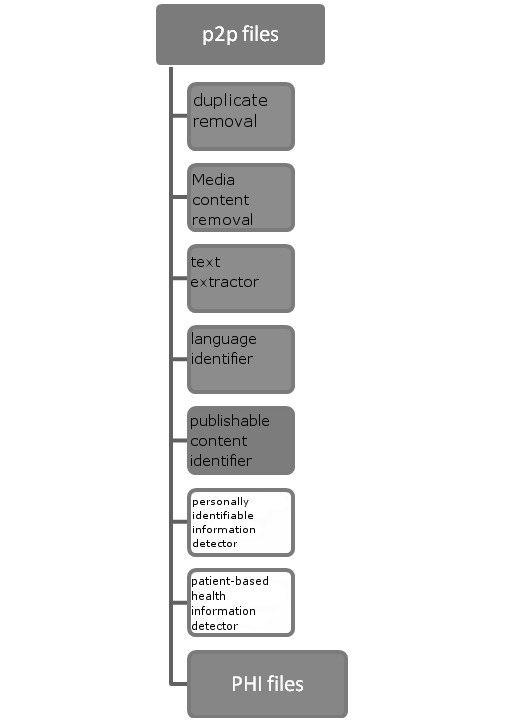
Components of P2P Watch. P2P = peer-to-peer, PHI = personal health information.

Figure 1 presents the system design.

#### Duplicate Removal

The first task of the file processing was to find and remove multiple copies of the same file; such duplicates can happen, as the same file can be harvested from multiple users. For each pair of files, we compared their sizes (in kilobytes), titles, and first and last sentences. If all the parameters were the same, we tagged two files as duplicates and kept only one for further processing.

#### Media Content Removal

We assumed that any published text was not leaking PHI—for example, writings describing fictional characters, and magazine and newspaper articles that contain information that is already public. We used Amazon Web Services as a source database of publication titles [25]. Although the number of titles fluctuates almost daily, the database has 400,000 to 500,000 titles for books; recording companies such as Sony, EMI, and Universal have more than 250,000 music titles in the database. Files with exact matching titles were discarded. Exceptions were made for files with titles that included such words as *notification*, *affidavit*, *justice*, *discharge*, and *lab*. Our system did not discard these files and retained them for further processing. If there was no exact title matching, the file was passed on for further processing.

#### Text Extractor

The text extractor converted files into raw text by removing all formatting meta-information. It also discarded nontext files (eg, images and music), corrupted files, and viruses. A wide range of input file formats suggested the use of several tools, with each tool extracting text from specific formats. We used the open source program Antiword to extract text from Microsoft Word documents [26]. To extract text from PDF, RTF, HTML, or XML files, we used the open source programs MineText [27] and GetText [28].

#### Language Identifier

We differentiated between English-language texts and texts written in other languages. We applied a publicly available language identifier, TextCat Language Guesser, which can identify 69 languages [29]. For text, the tool outputs several possible languages. If English was the most likely language of the text, then it appeared at the beginning of the output. Our manual examination had shown that, in our sample data, the first English tag always correctly marked texts written in English. We discarded a file if English was not the most likely language of the text.

#### Publishable Content Removal

P2P Watch looked for files with nonpersonal content. It filtered out published and educational materials (eg, assignments and theses) and nonpersonal texts (eg, manuals and technical reports) that were not found by the title lookup. We also hypothesized that music lyrics, discussion of popular fictional characters or current political events, and advertisement would be unlikely candidates for leaking explicit, detailed PHI. We built a list of fictional characters (eg, Bart Simpson), celebrities (eg, Paris Hilton), and public figures (eg, George Bush). We considered that, by the nature of their occupations, celebrities and public figures would have a lot of information about them publicly known and therefore any PHI pertaining to these individuals would not be considered a breach. To perform this task, we built a list of terms that appeared in the preface of publishable and educational texts; the terms are listed in Multimedia Appendix 1. We used regular expressions to locate those terms and their variations in the first 200 words of the file. Table 1 lists word categories and examples.

**Table 1:** Publishable content identifiers.

Category	Example
Books	Ebook, ISBN
Education	Thesis, assignment
Retail	Tim Hortons
Periodical	Magazine, article
Fictional	Harry Porter
Politics	Nicolas Sarkozy

#### PII Detector

**Figure 2:**
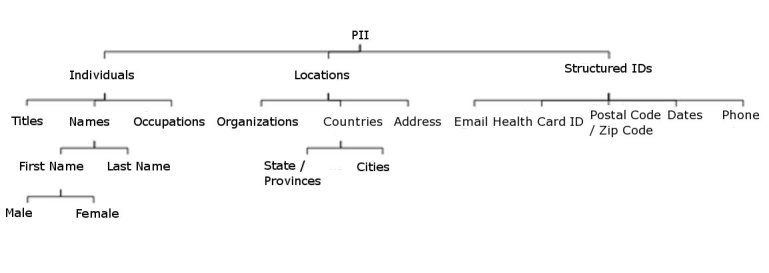
Personally identifiable information (PII) categories and their subcategories. ID = identification.

We considered that a person can be identified from first and last names, addresses, dates (which can be linked to identifiable events such as birth, death, and marriage), and organization names (eg, school, church, and professional association). We divided PII into three categories: person information (eg, names, family relations, and age-defining events), structured information (eg, dates, telephone numbers, and email), and geographic location (eg, street address and organization). For instance, telephone numbers are both geographic identifiers and numeric identifiers. Figure 2 illustrates the category relations.

For the personal name lookup, we acquired a female first name list, a male first name list, and a last name list. The lists contained both formal and informal name forms (eg, William, Bill, and Billy) and non-Anglo-Saxon names (eg, Meehai and Leila), as well as common misspellings (eg, Bll). The lists came with a commercial database marketing tool that performed best in an independent evaluation [30].

To reduce computationally expensive person name lookups, we first searched for patterns of family relations in the text (eg, “my daughter” and “an uncle of”), self-identification (eg, “my name” and “sincerely”), or life event (eg, “was born” and “died in”). Depending on the patterns, either the preceding or the following capitalized words were stored in a file’s name list. Having a file’s name list considerably accelerated the file processing. Further, when P2P Watch checked for a person name, it first checked with the file’s name list.

Additionally, PII included standardized information as follows: (1) telephone numbers: we looked for complete and incomplete formats, used in North America, (2) health insurance numbers: we looked for health insurance numbers assigned by each province (Canada), (3) dates: we restricted the dates to the 20th and 21st centuries, as earlier dates are unlikely to be related to health information of living human beings; also, dates had to be specific: “March 9th, 1999” indicated a specific date, whereas “March was chilly” did not, (4) email address: for example, john@canada.ca, john AT Canada DOT ca, (5) postal codes in Canada and zip code in the United States. These five categories were retrieved by manually built soft regular expressions.

Apart from geographic locations in Canada and the United States, we used some international information and introduced different granularity for different geographic categories. First was country: all the UN-recognized countries and their capitals (eg, France and Paris; Liberia and Monrovia) and self-proclaimed entities (eg, Eritrea and Abkhazia). Second was place. In the United States, we used state name, state capital, and—to cover the biggest single population unit—the largest city in the state; for example, we had Illinois, Springfield, and Chicago. In Canada, we used province, provincial capital, largest cities, and tourist attractions (eg, Alberta, Edmonton, Calgary, Banff), and the same for territories. In Europe, Latin America, Asia, Africa, and Australia, we used major cities that are not national capitals. The list is given in Multimedia Appendix 2.

The Canadian Judicial Council further considers certain organizations to be part of PII [31], such as public institutions (eg, schools and churches), care providers (eg, aid societies and foster homes), the names of support organizations (eg, women’s and senior’s support centers), and other location identifiers (eg, educational institutions and military bases). The organization names were expressed in many language forms. We modeled language patterns (eg, “lived in” and “come from”), organization types (eg, schools, military units, and churches), and the target population (eg, youth, women, and seniors).

To be marked as a text with PII, the file had to contain a geographic identifier and two other personal identifiers, such as a person’s first name and last name, or a person’s first name and date of birth. All the files marked as PII were passed on to the last component.

#### Patient-Oriented Health Information Detector

Disease names (eg, arthritis and mumps) and symptoms (eg, chest pain and headache), and procedures (eg, heart surgery and x-rays) most directly convey health information that is usually discussed in a clinical setting. To build a list of corresponding terms, we used the International Classification of Diseases [32] and the Medical Dictionary for Regulatory Activities (MedDRA) [33]. Drug names, too, may allow one to infer a specific medical, behavioral, or psychological condition or ailment of another individual. We used the Canadian Drug Product Database (Active and Inactive), which contains the names of drugs approved for use in Canada and previously available drugs [34]. To accommodate extraction of various drug names, we obtained a list of generic drug names and the trade names associated with them [35]. However, the resources listed above leave some gaps in the detection of health information. The most noticeable are acronyms (eg, ICU), specialties of health care providers (therapist, surgeon), and some condition names (blood pressure, tube fed). To fill the gaps, we manually searched Webster’s New World Medical Dictionary [36]. We minimized the above-mentioned resources by removing unrelated categories (animal diseases, animal drugs). Then the remaining texts were converted to lowercase, punctuation marks and numbers were removed, and stop words (eg, of and when) were eliminated. The list of the resulting keywords is given in Multimedia Appendix 3.

More details on the method can be found in Sokolova et al [19].

### Empirical Evaluation

Project approval from the Research Ethics Board of Children’s Hospital of Eastern Ontario was obtained prior to retrieving data from the P2P networks.

#### Files Analyzed

The files were gathered from April 2008 to June 2009 from three networks. We selected FastTrack, Gnutella, and eD2K networks due to their global popularity and high share of users [2]. To automatically search for and download P2P files, we modified the publicly available Shareaza P2P client [37], which is a software package allowing one to connect to multiple P2P networks simultaneously. In-house modifications to Shareaza included changes to the search function and an increase of logging capabilities.

We focused on capturing the most popular document formats: Microsoft Word (.doc), raw text (.txt), Rich Text Format (.rtf), Excel (.xls), PowerPoint (.ppt), Portable Document Format (.pdf), Extensible Markup Language (.xml), and HyperText Markup Language (.html). The search function was modified to automatically search for those formats and automatically retrieve the files. Automatic searches were conducted by the code at 15 minute intervals.

In total, we have gathered 3924 files. The data were sent for processing as is, without preliminary normalization: we preserved all the initial spelling, capitalization, grammar, and so on.

### Performance Evaluation

We evaluate the system’s efficiency through the time (seconds) it took each component to process the related files. Technical specifications of our equipment were as follows: Windows Server 2003 (Microsoft Corporation, Redmond, WA, USA), 3.20 GHz Intel Core i3 processor (Intel Corporation, Santa Clara, CA, USA), 4 GB RAM, and 500 GB SATA hard disk.

### Effectiveness Evaluation

We evaluated the effectiveness of P2P Watch by the number of PHI files found among the files it discarded as non-PHI (false-negative PHI files) and by the number of PHI files it marked as PHI (true-positive PHI files).

Exact estimation of true-positive PHI files was feasible due to the small output we expected. However, the exact estimation of false-negative PHI was not feasible due to the large volume of data. To estimate the presence of false-negative PHI files in the discarded files, we employed a sampling technique. The sampling technique is described below.

### Comparison With Other PHI Detection Tools

Currently deployed PHI tools are designed to analyze electronic health records produced by selected health care organizations [4-10]. The tools work on the assumption that (1) there are PHI words within each document, and (2) these words belong to a restricted number of categories (eg, local doctors, local hospitals). Most of the tools are proprietary and cannot be easily assessed for comparative evaluation [4-7,9,10].

The open source PHI detection tool De-id is popular among researchers [8,24]. To ensure a fair tool comparison and adherence to a main content assumption of PHI presence in the text, we applied De-id on P2P files that P2P Watch had been identified as PHI.

### Sample Size to Detect PHI in Discarded Files: False-Negative Evaluation

Because there were 3924 files in total and we expected most of them not to contain PHI, a manual examination of all of the discarded files would have been exceedingly time consuming. To compute the false-negative rate, we estimated the number of PHI files that could appear in a random sample of P2P files.

We wanted to determine sample sizes in advance. To obtain a conservative estimate, we decided on multiple sampling, where an individual sample is randomly drawn from a separate group of discarded files. Based on the P2P Watch architecture, we identified the three main groups of discarded files: group 1—files discarded by Amazon search, text extractor, and language identifier; group 2—files discarded by the content filter; group 3—files discarded by the PII detector and health information detector.

**Figure 3:**
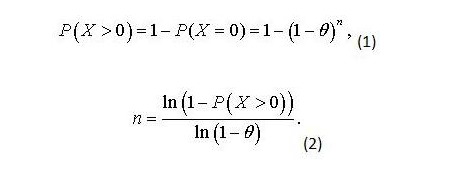
Equation (1, rearranged in 2) for determining the probability of detecting at least one file containing personal health information.

We used a binomial distribution as a model for P2P file data, assuming that either a file contains PHI or it does not [38,39]. Equation 1 in Figure 3 shows the probability of detecting at least one file with PHI, assuming a binomial distribution, where *θ *is the rate of PHI we wished to detect, and *n *is the number of independent samples. Equation 1 can be rearranged as Equation 2 (Figure 3).

Previous studies showed similarity in the ratio of PHI files among reviewed P2P files: approximately 1% of all the files contained PHI. Therefore, we used this estimate in Equation 2 to define the sample size for groups 1–3. For each group of discarded files, to have a 95% chance of detecting PHI when the underlying rate of files with PHI is at least 1%, we needed to sample at least 300 files. For the complete data, we wanted to sample at least 900 files.

### Special Protocols

Three special protocols were put in place for this study. First, we expected some files to contain inappropriate or obscene material (eg, pornography). We therefore did not explicitly look through image files (file extensions .gif, .jpg, .psd, .tif, and .bmp). Second, if we discovered any illegal materials (eg, child pornography), we passed that information on to the police. Third, if there were cases of disclosure of particularly sensitive personal information or PHI for a large number of individuals, then we reported them to the appropriate federal or provincial privacy commissioner for follow-up.

## Results

We applied P2P Watch for PHI detection in 3924 files exchanged on the three P2P networks. The total data set size was 9887 MB. The total processing time was 4132.41 seconds.

**Figure 4:**
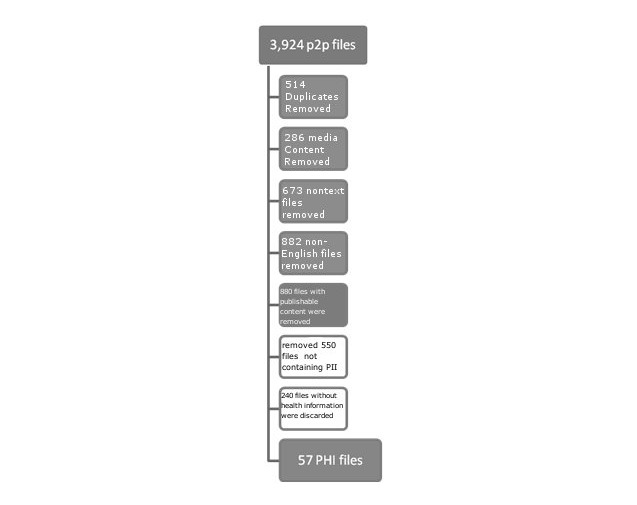
File processing steps by P2P Watch. PHI = personal health information, PII = personally identifiable information.

Figure 4 illustrates changes in the number of files processed by each component during our empirical evaluation.

### Observations

We made several general observations during the analysis as follows.

#### Duplicate File Removal

We considerably reduced processing time by first testing whether a file was a duplicate of an already gathered file. Removal of duplicates does not affect the quality of PHI detection, as P2P Watch should process the original file. We discarded 514 files (eg, Quick Recipe And Meal Ideas, 36 Christmas Carols & Songs), mostly books and technical manuals; the size of discarded files was 786 MB.

#### Media Content Removal

We discarded 286 files, which contained content that was found in the Amazon.com database (eg, Iron Crypt of the Heretics; The Haunted Lighthouse; New York, New York; ABBA’s The Winner Takes It All; and The Abominable Snowman); the size of discarded files was 1234 MB. We requested the exact match of the file title with the database entry; hence, some files with published content were passed through this filter.

#### Text Extractor

We worked with different file formats to extract text and discard images, virus files, and corrupted files. At this stage, 673 files were discarded, mostly mp3 and rm files with music and video contents; the size of the discarded files was 3077 MB.

#### Language Identifier

We identified English-language texts among the remaining files; 882 non-English texts were discarded, with a total size of 10 MB. To avoid misrepresentation of North America as a polyglot continent, we checked the discarded files and found that many of them had explicitly erotic content.

#### Publishable Content Identifier

Based on processing of the first 200 words, 880 files were discarded, with a total size of 171 MB. Books composed the majority of the discarded files, with the remaining part comprising cover letters, resumes, homework, and forms.

#### Personally Identifiable Information Detector

Based on the search for PII within a complete file, 550 files were discarded. Most of these texts were in a small publishable form such as articles, opinion pieces, local community letters, forms, and job application packages. The size of discarded files was 3.9 MB.

#### Patient-Oriented Health Information Detector

The last component identified 57 files as potential PHI and discarded 240 files. Among the discarded files, most promoted different types of consumer goods and services (credit services, fitness and skin care, gadgets, etc); some tax and insurance forms were discarded as well.

### Efficiency Evaluation

**Figure 5:**
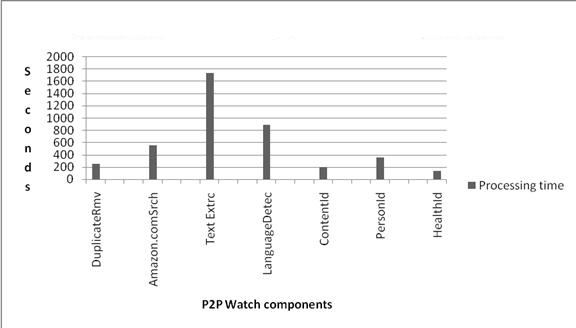
Processing time (seconds) of P2P Watch components.

We timed the performance of each component. The most time was used by the text extractor, the first component to process the complete file text. The most efficient components were duplicate removal and Amazon.com search, which processed the most files in the least time. Figure 5 illustrates time spent by each component on the data processing.

### Effectiveness Evaluation: True-Positive Rate

P2P Watch flagged 57 files as containing PHI. Manual analysis confirmed that all the files contained PII and PHI. However, we distinguished between true PHI and pseudo-PHI. Among these 57 files, 11 contained health information about an identifiable individual: they were indeed PHI (true PHI). PHI appeared in various types of documents—for example, a note to a temporary guardian and a lawyer’s note were shown to contain PHI.

Other marked files contained both PII and PHI, but were not PHI (pseudo-PHI). A few examples of pseudo-PHI discovered through a manual check of the P2P files contained information that the information owner had intentionally allowed to be part of a public audience. In one instance, the data owner stated “When I was four, I ended up in the hospital for playing with medicine” as part of a book report on Curious George. Another pseudo-PHI file was a nursing student’s assignment, which contained initials for a patient at a hospital and a room number, but not enough information to identify the patient without access to more detailed hospital records. The curriculum vitae of a medical professional and a medical insurance form are other examples of such files.

### Effectiveness Evaluation: False-Negative Rate

We ran P2P Watch without the duplicate removal filter. We invited two independent evaluators to read all the sampled files and mark them as PHI or not. One author (EN) participated in the evaluation of some files.

We first randomly selected three samples of 300 discarded files, where one sample consisted of files discarded by Amazon search, text extractor, and language identifier; another sample consisted of files discarded by the content filter; and the third sample consisted of files discarded by the PII detector and health information detector.

None of sampled 900 files contained PHI. We, however, were concerned with the possibility of PHI in files discarded by the PII detector and health information detector. We therefore chose to manually check all the remaining 678 files in this stratum, and found none containing PHI.

As a result, we manually checked 1578 discarded files and found that none of them contained PHI.

### Comparison With De-id

The open source tool De-id [8] gave us an opportunity to test the applicability of existing tools to finding PHI in P2P files. We applied De-id to find PHI in the 57 files that P2P Watch marked as PHI. By that time, we knew that the files indeed contain PII and PHI. We had to use the text extractor component, as De-id works only with text format.

De-id crashed on 3 files. The tool did not recognize any of remaining 54 files as PHI. The tool mislabeled many critical terms (eg, *risk *and *blood *were both marked as ambiguous last names, and *disorder *and *depression *were not recognized as health related). De-id took an average of 11 seconds to process a short file.

This empirical evidence showed that major components have to be added to De-id: a text extractor, and new PII and PHI detection components.

## Discussion

In this study, we have introduced P2P Watch, which detects PHI in files shared by users of P2P networks. Albeit the proportion of PHI files among P2P files is rather small, the overall problem is big, as by some estimates 50% of files downloaded and 80% of files uploaded on the Internet are through P2P networks[40]. However, even one PHI file can do much harm, especially if it contains an exact pointer to a publicly available data base. At the same time, we empirically showed that traditional de-identification tools are not designed to detect PHI in P2P files.

P2P Watch is capable of working within the complex environment of P2P networks. It detects PHI in files in which context, content, and format type vary. Within the data set of 3924 P2P files, the system detected 11 PHI files. Our manual evaluation of 1578 files, marked by the system as non-PHI, confirmed that these files indeed did not contain PHI. The sampling results showed that P2P Watch was very unlikely to miss PHI files.

For successful PHI detection in P2P networks, it is essential that the detection system process large volumes of heterogeneous data input in a timely manner and can withstand substantial irrelevant information. A reliable solution is based on two factors [41]. First, a high confidence that the limited number of analyzed texts will not exclude any possible PHI texts is needed; this can be achieved through filtering out only the files guaranteed not to contain PHI. Second, a speedy detection process is needed that prevents a prolonged presence of PHI texts on the network; this can be achieved through minimizing time of filtering with respect to performed text analysis.

P2P Watch efficiently reduces the time of PHI exposure. Our detection strategy is up-front shallow text processing, whose goal is to quickly process the vast majority of input files, followed by a thorough analysis of a small number of selected texts. This thorough analysis phase used electronic and hard-copy dictionaries of health care terms, an ontology of medical terms, and lists of personal names. We supplemented those sources with in-house built gazetteers (topical lists of geographic information) and lists of organization types.P2P Watch reserves comprehensive text analysis for a small number of selected files, while performing fast and accurate shallow processing for the vast majority of files. This is the principal difference from previously built systems, which process all the input files equally. The difference may be explained by the fact that previous systems were designed to detect patient’s PII in electronic medical records, whereas our P2P Watch searches for both PII and PHI in previously unseen documents. Once a file is flagged as containing PHI, the individuals affected can be alerted. A search for a data custodian’s name within the flagged files would indicate which custodian to alert, for example.

Several possible expansions of the functionality of P2P Watch are being considered for future work. Our current detection is limited to text written in English. Expanding P2P Watch capacities to other language such as French and Spanish would capture more PHI leaks. Furthermore, we want to build separate components to identify files that contain PII and HI, but are not PHI (pseudo-PHI). Resumes, recipes, incomplete health forms (eg, insurance), and public health announcements are examples of files that were falsely labeled as PHI. The idea would be to detect these types of documents and automatically exclude them early in the P2P Watch pipeline. A challenge with forms is that the empty forms may contain pseudo-PHI (eg, fields for human immunodeficiency test results). Special analysis of such forms is required to determine which content is part of the form and which is completed by the user.

Another future avenue is to add localized detection of PHI in the United States; this expansion may involve building new customized lists of organization and trademark names. In the future, a deeper analysis phase, perhaps coreference resolution, could be done, potentially increasing the precision of the whole detection process.

## Multimedia Appendix 1

Publishable information keywords.

## Multimedia Appendix 2

Geographic locations.

## Multimedia Appendix 3

Health information keywords.

## Abbreviations

P2P: peer-to-peer
PHI: personal health information
PII: personally identifiable information
